# Psychological treatments for depression among women experiencing intimate partner violence: findings from a randomized controlled trial for behavioral activation in Goa, India

**DOI:** 10.1007/s00737-019-00992-2

**Published:** 2019-07-30

**Authors:** Anushka Rajesh Patel, Benedict Weobong, Vikram Harshad Patel, Daisy Radha Singla

**Affiliations:** 1 Department of Psychology, The University of Tulsa, 800 S. Tucker Drive, Tulsa, OK 74104 USA; 2 School of Public Health, Department of Social and Behavioural Sciences, College of Health Sciences, University of Ghana, Accra, Ghana; 3 Department of Global Health and Social Medicine, Harvard Medical School, 641 Huntington Avenue, Boston, MA 02115 USA; 4 Sangath, H No 451 (168), Bhatkar Waddo, Socorro, Bardez, Porvorim, Goa 403501 India; 5 Department of Psychiatry, University of Toronto and Sinai Health System, 600 University Ave, Rm914A, Toronto, Ontario M5T 1R8 Canada

**Keywords:** Depression, Intimate partner violence, Behavioral activation, India, LMIC

## Abstract

Intimate partner violence (IPV) strongly predicts depression, but it is unknown if women experiencing IPV can benefit from depression treatments in contexts where depression and IPV are prevalent. This study explored whether women experiencing IPV in Goa, India, can benefit from the Healthy Activity Program (HAP), a culturally adapted behavioral activation treatment, compared with enhanced usual care (EUC). Cross-sectional and longitudinal analyses were performed on data from a clinical trial. Measures assessed at baseline and 3 and 12 months included depressive symptoms. Measures assessed at 3 and 12 months included activation and IPV. Independent *t* tests were conducted to assess if participants experiencing IPV had higher depressive symptoms and lower activation at 3 and 12 months; hierarchical linear regression was conducted to determine if 3-month IPV predicted 12-month depressive symptoms across trial arms (Hypothesis 1). Hierarchical linear regression was then conducted to examine if the relationship between 3-month activation and 12-month depressive symptoms was moderated by 3-month IPV within each trial arm (Hypothesis 2). As expected, participants experiencing IPV had significantly lower activation levels and higher depressive symptoms compared with participants who did not experience IPV at 3 and 12 months in cross-sectional analyses. Similarly, IPV endorsed at 3 months significantly predicted depressive symptoms at 12 months. However, activation was significantly associated with less severe depressive symptoms at 12 months, irrespective of IPV endorsement among HAP participants. For EUC participants, IPV remained the only significant predictor of depressive symptoms at 12 months. Results suggest that women experiencing IPV can still benefit from behavioral activation.

**Electronic supplementary material:**

The online version of this article (10.1007/s00737-019-00992-2) contains supplementary material, which is available to authorized users.

Intimate partner violence (IPV), physical or sexual violence perpetrated by a current or former partner, is a global epidemic (World Health Organization; WHO 2013). Rates of IPV are high in low- and middle-income countries (LMICs) (Halim et al. 2018), including India, where married women of childbearing age report the highest rates of IPV. For example, 37.3% of South Asian women (WHO 2013) and 49% of Indian women (Dalal and Lindqvist 2012) report IPV. Intimate partner violence is a consistent risk factor for depression (Devries et al. 2013), which is the leading cause of disability among women of childbearing age (Ferrari 2013). Designing and evaluating interventions for depression among IPV survivors is needed. We conducted secondary analyses to explore whether and how IPV impacted depressive symptoms following the Healthy Activity Program (HAP)—a brief culturally adapted behavioral activation treatment, which effectively reduced depressive symptoms at post-treatment (Patel et al. 2017) and 1-year follow-up (Weobong et al. 2017).

## Prevalence, impact, and nature of IPV in India

India is a patriarchal country with historically high rates of IPV (Simister and Mehta 2010). The Indian National Family Health Survey found that 49% of 124,385 ever-married women experienced physical or sexual IPV (Dalal and Lindqvist 2012). Medical consequences of IPV include sexually transmitted diseases (Mahapatro et al. 2012; Silverman et al. 2008), gynecological complications (Raj et al. 2011), and child malnutrition and mortality (Duvvury et al. 2013; India State-Level Disease Burden Initiative Suicide Collaborators et al. 2018). Psychological consequences include post-traumatic stress disorder (PTSD) and common mental disorders (e.g., depression and anxiety). For instance, 14% of women who experienced IPV in an outpatient psychiatric treatment-seeking sample in India reported PTSD and almost all (99%) met the criteria for depression (Chandra et al. 2009; Varma et al. 2007). Similarly, a community-based cross-sectional study found that 22.3% of Indian women experiencing IPV reported suicidal ideation and 21.3% reported common mental disorders (Vachher and Sharma 2010).

In India, IPV disproportionately affects people with less education, from marginalized castes, who live in poverty (Ackerson and Subramanian 2008) and are gender disadvantaged (Patel et al. 2006). As 22% of Indians live in poverty (The World Bank 2016), a sizeable segment of the population is at risk of IPV. Additionally, IPV is a culturally sanctioned form of violence against women (Dalal et al. 2012; Nakray 2013) and may be compounded by sociopolitical and legal barriers against seeking help (Rege 2011; Tichy et al. 2009).

## The role of psychological treatments for populations experiencing IPV

Multiple evidence-based depression treatments have been disseminated in LMICs (Singla et al. 2017). Yet, it is unclear how ongoing IPV—a consistent risk factor for depression (Devries et al. 2013)—impacts treatment outcomes. The effectiveness of depression treatments among high-risk sub-groups experiencing IPV warrants investigation, as they may not fully benefit from depression treatments due to comorbidities such as PTSD, anxiety, and suicidal tendencies (Dutton et al. 2006; Pico-Alfonso et al. 2006). Unfortunately, the evidence is not consistent because treatment studies routinely exclude people experiencing IPV as they pose safety risks (Poleshuck et al. 2016). Failure to include IPV survivors or assess IPV in treatment studies contradicts best practices of screening, safety planning, and case management (Morse et al. 2012). Safety planning around IPV in LMICs is feasible. Murray et al. (2014) demonstrated safety planning with IPV survivors in Southern Iraq and the Thailand/Burma border through engagement with multiple staff, 24-hour watch by doctors, collaborations with local organizations, and in-person and phone supervision from local counselors. With the exception of one study that incorporated safety planning and used interpersonal therapy for depression for IPV survivors (Cort et al. 2014), such practices are not commonly implemented (Murray et al. 2014). The lack of systematic IPV assessment limits a broader understanding of how depression treatments fare among such high-risk sub-groups.

### Behavioral activation in populations experiencing IPV

Behavioral activation (BA) is an evidence-based psychological treatment for depression (Dimidjian et al. 2006; Dobson et al. 2008). In the BA model, depression is perpetuated by withdrawal behaviors. Individuals engaging in BA break the cycle of depression by activating their behaviors to draw pleasure and mastery from their environment (Dimidjian et al. 2011). Although treatments that improve activation have been widely disseminated (Dimidjian et al. 2011), studies have not considered if BA can improve depressive symptoms among women experiencing IPV. Improving activation among women experiencing IPV may be particularly challenging because activation may be curtailed by IPV. Specifically, partners who perpetrate IPV also limit their partners’ access to social support, medical autonomy, financial and decisional freedom, and mobility (Dutton and Goodman 2005; McCloskey et al. 2007). These restrictions may isolate women from opportunities for non-depressive activities and hinder their ability to draw pleasure and mastery from their environment. As activation is the putative mechanism of change in BA, hindering activation could influence treatment response overall. Conversely, individuals who experience IPV and depressive symptoms may benefit by accessing more pleasure and mastery to offset the psychological risks of IPV. Weobong et al. (2017) found that activation mediated the relationship between trial arm and depressive symptoms at 12-month follow-up, confirming that activation was the mechanism of therapeutic change. This study sought to examine if activation was susceptible to suppression by IPV through a moderation analysis and if this moderation differed for women who received enhanced usual care (EUC) or HAP.

## Study hypotheses

We examined the individual and interactive roles of IPV and activation on depressive symptoms between an active treatment group (i.e., HAP) and control group (i.e., EUC) from a clinical trial in Goa, India. Consistent with the literature linking IPV with depressive symptoms, we explored the following hypothesis:

1. Hypothesis 1: IPV will relate to lower activation and higher depressive symptoms compared with participants who do not report IPV at 3- and 12-month post-enrollment; IPV and activation at 3-month post-enrollment will predict depressive symptoms at 12-month follow-up. This hypothesis was tested in order to lay the foundation for testing a moderation effect in Hypothesis 2.

If IPV and activation individually predict depressive symptoms at 12-month follow-up, then we will explore a possible moderator by which IPV may limit treatment gains.

(2) Hypothesis 2: IPV will moderate the relationship between activation at 3-month post-enrollment and depressive symptoms at 12-month follow-up. Specifically, activation will be reduced for participants experiencing IPV regardless of the trial arm.

## Materials and methods

The HAP treatment was evaluated as part of the larger Program for Effective Mental Health Interventions in Under-resourced Health Systems (PREMIUM) study (Patel et al. 2014; Patel et al. 2017) among adult primary care attendees in Goa, India. We examined whether participants who experienced IPV could still have reduced depressive symptoms depending on whether they were administered HAP vs. EUC with cross-sectional and longitudinal analyses.

### Participants

**Fig. 1:**
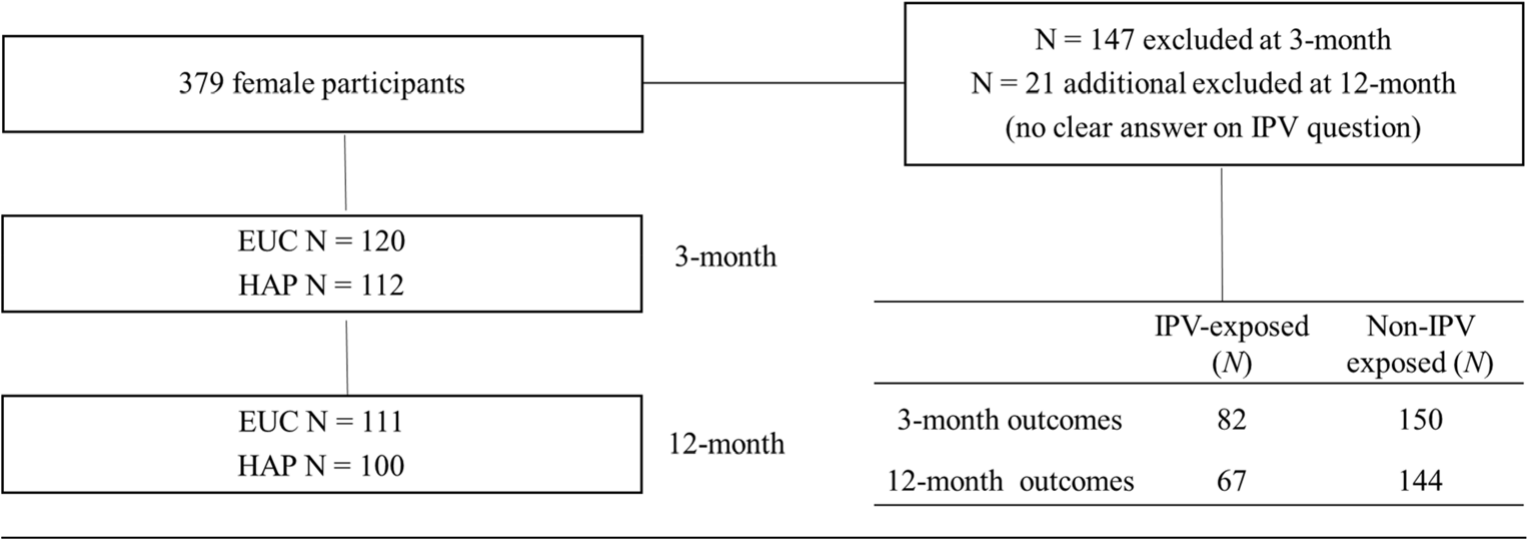
3-month post-enrollment analyses had total *N* = 232, whereas 12-month follow-up analyses had *N* = 211 for independent samples *t* tests. However, *N* = 220 for regression analyses after accounting for list-wise deletion procedures, wherein the regression software deletes any cases with missing data

Participants were a sub-sample of married women randomized to HAP or EUC who completed assessments at baseline and 3 and 12 months. For details on randomization and enrollment procedures, see the full paper by Patel et al. 2014. Figure 1 delineates how the sub-sample was selected. Married women were selected because IPV in India is directed towards women rather men (Nakray 2013). Participants who were enrolled in either HAP or EUC and clearly endorsed a yes or no answer for IPV were included. IPV survivors dropped out of treatment at similar rates to women who did not experience IPV (29.41% vs. 20.51%, respectively). Anti-depressant medication use was equivalent between trial arms throughout the course of treatment (i.e., 4% for both EUC and HAP) (Patel et al. 2017).

### Trial arms

#### Healthy Activity Program

The HAP treatment was a culturally adapted BA. It was delivered over 2 to 3 months in six to eight 30–45 min sessions. The core interventions of HAP included psychoeducation on activity and mood, behavior monitoring, activity scheduling, social network activation, and problem solving. Lay counselors who underwent a 3-week training in HAP and satisfied competency requirements during the trial’s pilot phase delivered the treatment (Singla et al. 2014).

#### Enhanced usual care

Primary care providers were equipped with additional resources to enhance the usual care condition (Patel et al. 2014). Providers were given screening results of participants with clinically elevated depressive symptoms, a contextualized copy of the mhGAP guidelines, and information about making referrals for psychological care. However, refractory cases were referred for specialist care if their symptoms did not improve during treatment or at 1-month post-treatment.

### Measures

Measures were collected at baseline and at 3- and 12-month post-enrollment. A demographic measure (assessing gender, age, religion, and occupation) and measures of depressive symptoms were administered at baseline, 3-month post-enrollment, and 12-month follow-up. Measures of activation and IPV were only administered at 3-month post-enrollment and at 12-month follow-up.

### Dependent variable

#### Depressive symptom severity

Depressive symptom severity was measured using the Patient Health Questionnaire-9 (PHQ-9; Kroenke et al. 2002), a 9-item measure with a 4-point (0–3) Likert scale. This questionnaire queries all depressive symptoms against response options of “not at all,” “several days,” “more than half the days,” and “nearly every day” in the past 2 weeks.

### Independent variables

#### Intimate partner violence (IPV)

The experience of IPV was estimated by administering a 2-item questionnaire to all participants. Participants were asked the following two questions: (1) “Sometimes husbands/wives get angry and abuse their partners. In the past three months, has your husband/wife ever spoken to you using language which is threatening (e.g., that he/she was going to hit you) or abusive (e.g., called you names, accused you of having relations with other men/women etc.)?” and (2) “In the past three months, has your husband/wife slapped, hit, kicked, pinched, pulled your hair?” Answers such as “I don’t know” or “I refuse to answer” were also noted. Using these questions, IPV was scored as a categorical variable to create groups. Participants endorsing any form of IPV (psychological, physical, or both) in the past 3 months were considered participants experiencing IPV (scored “1”). The remaining participants were considered non-IPV-exposed (scored “0”). The decision to combine IPV types was grounded in research that psychological and physical forms of IPV typically co-occur (Coker et al. 2000) and each type of IPV is associated with low self-esteem and suicidal thoughts, which are also symptoms of depression (Ellsberg et al. 2008).

#### Activation

Activation was measured using the self-report Premium Abbreviated Activation Scale (PAAS; Weobong et al. 2017). This abbreviated scale contained 5 items rated with a 4-point Likert scale. Scores for activation ranged from 0 to 20.

### Potential covariates

Potential covariates included demographic characteristics.

### Analytic strategy

All analyses were conducted using SPSS 22.0 (IBM Corp 2013). Means and standard errors of all variables were computed on relevant demographic and clinical characteristics (see Table 1). Correlations between relevant baseline, 3-month, and 12-month variables were computed to determine which variables were significantly associated with the dependent variable (i.e., depressive symptoms at 12-month follow-up), to control for in regression models (see supplementary tables).

**Table 1:** Demographic and clinical characteristics of women collected at baseline by the trial arm

Category	HAP (%)	EUC (%)
	*N* = 112	*N* = 120
Demographic characteristics		
Age		
Religion		
Hindu	93.8	91.7
Christian	0.9	5.8
Muslim	5.4	2.5
Educational status		
No education	32.1	27.5
Primary complete	47.3	51.7
Secondary complete	17	15
Higher secondary complete	2.7	4.2
Graduate	0.9	1.7
Occupation		
Student	75	71.7
Unemployed	20.5	27.5
Housewife	0.9	0.8
Laborer	3.6	0
Clinical characteristics		
Variable	Mean (SE)	Mean (SE)
Baseline depressive symptoms	17.79 (0.25)	17.51 (0.22)
Age	42.73 (0.95)	42.11 (0.87)
3-month depressive symptoms	8.06 (0.70)	11.44 (0.64)
3-month PAAS	12.01 (0.43)	10.01 (0.38)
12-month depressive symptoms	8.19 (0.67)	10.61 (0.71)
12-month PAAS	10.9 (0.46)	10.03 (0.40)

#### Statistical tests

Independent samples *t* tests were conducted, combining trial arms, to analyze the first part of Hypothesis 1 that participants experiencing IPV will have lower activation and higher depressive symptoms compared with non-IPV-exposed participants in cross-sectional analyses at 3-month post-enrollment and 12-month follow-up. Next, a hierarchical linear regression was conducted, combining trial arms, to examine the second part of Hypothesis 1 that IPV and activation at 3-month post-enrollment will predict depressive symptoms at 12-month follow-up. Independent variables were entered into models to predict the dependent variable of depressive symptoms at 12-month follow-up. Specifically, baseline depressive symptoms and age were entered as covariates in model 1; IPV endorsed at 3-month post-enrollment and activation levels at 3-month post-enrollment were entered as predictors in model 2.

A hierarchical multiple regression analysis was conducted to test Hypothesis 2 by stratifying the dataset by the trial arm to separately examine predictors of treatment response. Hypothesis 2, which investigated the joint effect of IPV with activation (i.e., the moderator), was examined separately for each trial arm because activation differed significantly between trial arms (see Weobong et al. 2017). As activation demonstrated different ceiling effects in each trial arm, we examined Hypothesis 2 by stratifying the dataset into trial arms. Independent variables were entered into individual regression models to predict the dependent variable of depressive symptoms at 12-month follow-up. Specifically, baseline depressive symptoms, activation levels at 3-month post-enrollment, and age were entered as covariates in model 1; IPV endorsed at 3-month post-enrollment was entered as a predictor in model 2; and the interaction term (i.e., IPV at 3-month post-enrollment and activation at 3-month post-enrollment) was entered in model 3.

## Results

### Participant demographic characteristics

The total sample included *N* = 232 participants, which encompassed married women (*M*_age_ = 42.06, SD_age_ = 9.60), the majority of whom were Hindu (92.7%) housewives (73.3%), with a primary-level education through grade 8 (49.6%) (see Table 1). Further, IPV prevalence was reportedly 35.34% at 3-month post-enrollment and 31.75% at 12-month follow-up (see Fig. 1).

- Hypothesis 1: IPV will be associated with lower activation and higher depressive symptoms compared with participants who do not report IPV at 3- and 12-month post-enrollment; IPV and activation at 3-month post-enrollment will predict depressive symptoms at 12-month follow-up.

#### Cross-sectional analyses

**Fig. 2:**
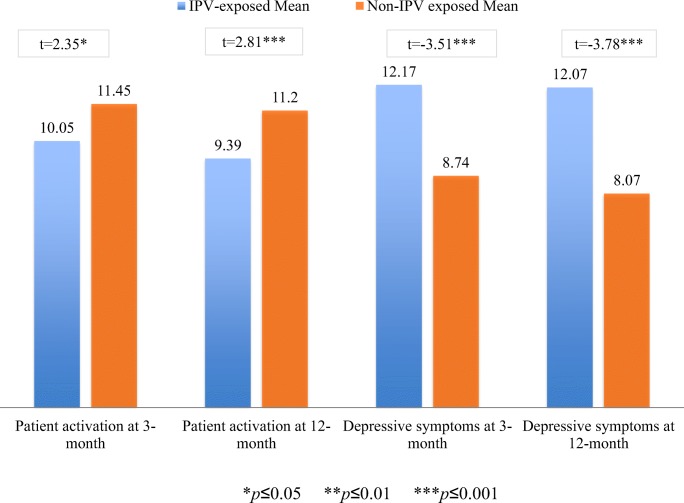
Independent samples *t* tests at 3-month and 12-month post-enrollment for activation and depressive symptoms, respectively

Results of the independent samples *t* test (*N* = 232) confirmed our prediction that participants endorsing IPV would have lower activation and higher depressive symptoms at both time points. Participants who endorsed IPV at 3-month post-enrollment had significantly lower activation levels compared with participants who did not endorse IPV. This difference demonstrated a small effect size (*d* = 0.32 (95% CI 0.05–0.59)). These results were replicated at 12 months (*d* = 0.42 (95% CI 0.12–0.71)). Similarly, participants who endorsed IPV at 3-month post-enrollment had significantly higher levels of depressive symptoms compared with participants who did not endorse IPV. The difference between groups demonstrated a medium effect size (*d* = 0.48 (95% CI 0.21–0.75)). These results were replicated for 12-month outcomes, indicating a medium effect size (*d* = 0.56 (95% CI 0.26–0.85), see Fig. 2).

#### Longitudinal analyses

Results of the hierarchical linear regression (*N* = 211) confirmed our hypothesis that experiencing IPV at 3-month post-enrollment would predict higher depressive symptoms at 12-month follow-up. The overall model was significant (*R*^2^ = 0.15, *F*(4, 215) = 10.36, *p <* .001). Higher activation (*β* = − 0.32; *p* < 0.001), IPV at 3-month post-enrollment (*β* = − 0.17; *p* = 0.008), and older age (*β* = 0.19; *p* = 0.004) significantly predicted higher depressive symptoms at 12-month follow-up. Our cross-sectional and longitudinal analyses held in the expected direction; not only were activation lower and depressive symptoms higher for women who reported IPV at 3-month post-enrollment, but IPV endorsement also predicted future depressive symptoms at 12-month follow-up. Having established the individual effects of activation and IPV across time, we proceeded to test whether IPV and activation at 3-month post-enrollment were interacting to influence depressive symptoms at 12-month follow-up.

- Hypothesis 2: IPV will moderate the relationship between activation at 3-month post-enrollment and depressive symptoms at 12-month follow-up. Specifically, activation will be reduced for participants experiencing IPV regardless of the trial arm.

Results of the regression analyses showed no support for our second hypothesis. Within HAP (*n* = 105), the final model was significant, explaining approximately 27% of variance in 12-month follow-up depressive symptoms for HAP participants (*R*^2^ = 0.27, *F*(1, 99) = 7.20, *p <* .001). However, IPV at 3-month post-enrollment did not moderate the relationship between activation at 3-month post-enrollment and depressive symptoms at 12-month follow-up (see Table 3). Within EUC (*n* = 115), the final model was not significant (*R*^2^ = 0.09, *F*(5, 109) = 2.41, *p =* 0.066). The interaction term was also not significant (see Tables 2 and 3).

**Table 2:** Regression analyses examining the role of 3-month IPV individually and as a moderator on 12-month depressive symptoms for EUC

Enhanced usual care arm (*N* = 115)																		
	Model 1						Model 2						Model 3					
	*R* square = 0.045 (ns)						*R* square = 0.08 (ns)						*R* square = 0.089 (ns)					
Predictors	*B*	Std. error	Beta	*t*	Sig	% unique variance	*B*	Std. error	Beta	*t*	Sig	% unique variance	*B*	Std. error	Beta	*t*	Sig	% unique variance
Constant	1.36	5.90		0.23	0.82		0.18	5.84		0.03	0.97		− 0.24	5.85		− 0.04	0.97	
Age	0.10	0.08	0.12	1.26	0.21	1.36	0.13	0.08	0.16	1.65	0.10	2.27	0.13	0.08	0.16	1.70	0.09	2.42
Baseline depressive symptoms	0.27	0.30	0.09	0.93	0.35	0.74	0.20	0.29	0.06	0.69	0.49	0.40	0.21	0.29	0.07	0.72	0.47	0.43
3-month activation	− 0.33	0.18	− 0.18	− 1.84	0.07	2.92	− 0.29	0.18	− 0.16	− 1.67	0.10	2.32	− 0.47	0.24	− 0.26	− 1.93	0.06	3.13
3-month IPV	N/A						3.01	1.46	0.19	2.06	0.04	3.55	3.43	1.51	0.22	2.27	0.03	4.29
3-month activation*IPV							N/A						0.36	0.34	0.14	1.06	0.29	0.94

**Table 3:** Regression analyses examining the role of 3-month IPV individually and as a moderator on 12-month depressive symptoms for HAP

Healthy activity program arm (*N* = 105)																		
	Model 1						Model 2						Model 3					
	*R* square = 0.25*						*R* square = 0.27*						*R* square = 0.27*					
Predictors	*B*	Std. error	Beta	*t*	Sig	% unique variance	*B*	Std. error	Beta	*t*	Sig	% unique variance	*B*	Std. error	Beta	*t*	Sig	% unique variance
Constant	0.03	4.95		0.01	1.00		− 1.19	5.00		− 0.24	0.81		− 1.16	5.02		− 0.23	0.82	
Age	0.14	0.06	0.20	2.32	0.02	4.00	0.15	0.06	0.21	2.39	0.02	4.19	0.14	0.06	0.20	2.33	0.02	4.03
Baseline depressive symptoms	0.16	0.24	0.06	0.68	0.50	0.34	0.19	0.24	0.07	0.79	0.43	0.46	0.19	0.24	0.07	0.79	0.43	0.46
3-month activation	− 0.73	0.14	− 0.47	− 5.42	< 0.0001	21.77	− 0.70	0.14	− 0.45	− 5.11	< 0.0001	19.18	− 0.67	0.15	− 0.43	− 4.32	< 0.0001	13.80
3-month IPV	N/A						1.86	1.31	0.12	1.42	0.16	1.48	1.89	1.31	0.13	1.44	0.15	1.53
3-month activation*IPV							N/A						− 0.15	0.34	− 0.04	− 0.45	0.65	0.15

*Note. None of the full models were significant (*p* > 0.05), but individual predictors in model 3 did explain significant variance. “*B*” refers to unstandardized coefficients in original units, “Std. error” refers to the standard error around unstandardized coefficients, “Beta” refers to standardized regression coefficients, “*t*” refers to the *t* statistic generated by the regression, and “Sig” refers to the significance value of the model and its constituent predictors, setting alpha at 0.05. “% unique variance” explains how much each predictor uniquely contributed to explaining variance in outcomes after controlling for others in the model

**Note. All models were significant (*p* < 0.001)*, but *F* change was only significant for model 1, suggesting that addition of variables did not improve incremental prediction. “*B*” refers to unstandardized coefficients in original units, “Std. error” refers to the standard error around unstandardized coefficients, “Beta” refers to standardized regression coefficients, “*t*” refers to the *t* statistic generated by the regression, and “Sig” refers to the significance value of the model and its constituent predictors, setting alpha at 0.05. “% unique variance” explains how much each predictor uniquely contributed to explaining variance in outcomes after controlling for others in the model

## Discussion and conclusions

This study explored relationships between IPV, activation levels, and depressive symptoms that have not yet been explored to our knowledge. Our findings reflect the wider literature in some respects. First, reported rates of IPV at 3-month post-enrollment (35.34%) and 12-month follow-up (31.75%) were comparable with other estimates from South Asia (37.5%) (WHO 2013). Our slightly lower IPV rates may reflect higher education levels of Goa compared with other states (Chandramouli and General 2011). Second, cross-sectional relationships between IPV, activation, and depressive symptoms held in the expected directions. We found significantly higher depressive symptoms among women experiencing IPV, a finding supported worldwide and in India (Maitra et al. 2015; Patel 2005; Pereira et al. 2007; Rao et al. 2012). We also found significantly lower activation among women experiencing IPV. To our knowledge, no studies have examined activation levels among women experiencing IPV. Our findings contribute to the literature by showing that women experiencing IPV are less activated and may, therefore, benefit from a more holistic treatment approach. For instance, transdiagnostic treatments, such as the common elements treatment approach (CETA), have been successfully implemented with individuals experiencing IPV and depression (Bass et al. 2013). Similarly, interpersonal therapy has improved depressive symptoms, functional outcomes, and PTSD among trauma survivors (Cort et al. 2014; Jiang et al. 2014), and it may be another promising treatment for IPV survivors.

In contrast to HAP participants, EUC participants’ depressive symptoms were only predicted by IPV. One explanation for differences between trial arms may be due to activation differences. Activation mediated the relationship between the trial arm and sustained clinical outcomes in the PREMIUM trial (Weobong et al. 2017), indicating that women who learned to activate their behaviors (i.e., HAP participants) had higher activation and lower depressive symptoms irrespective of IPV exposure. Indeed, women experiencing IPV still demonstrated higher activation levels and lower depressive symptoms following HAP compared with women exposed to IPV following EUC. Given that activation is the key mechanism of BA, increasing activation may have buffered against the psychological impact of IPV. For instance, women may have activated their behaviors to improve interpersonal supports, which has shown to mediate the effects of depression interventions on depressive symptoms (Singla et al. 2015). Similarly, women may have developed greater self-efficacy from activating their behaviors, which is linked with reduced depressive symptoms (Leahy-Warren et al. 2012).

Although women experiencing IPV had lower activation overall, our hypothesized moderator—an interaction between IPV and activation—was not supported. This finding was contrary to what we expected. However, the non-significant interaction suggests that women experiencing IPV can feasibly improve their activation levels and decrease depressive symptoms. Several treatment components of HAP could explain these outcomes. First, HAP inculcated the skills of monitoring, structuring, and scheduling activities. HAP also focused on problem solving, which is an empirically supported depression treatment (Bell and D’Zurilla 2009). Teaching women experiencing IPV how to problem-solve barriers to activation may partly explain why IPV did not suppress activation. Second, HAP taught women how to activate their social networks, and social support is a robust protective factor against depression (Panzarella et al. 2006) and IPV (Coker et al. 2004; Sylaska and Edwards 2014).

### Study implications

Overall, our study demonstrates the feasibility of treating depression for a high-risk sub-group that is typically excluded from depression trials (Poleshuck et al. 2016). Given that IPV remained a significant predictor of depressive symptoms for EUC participants, training primary care providers to assess and provide safety planning for women experiencing IPV in settings where IPV and depression are prevalent may improve clinical outcomes (Murray et al. 2014). Additional studies are required to determine if treating depressive symptoms may impact other IPV-related outcomes, such as future IPV re-victimization. In an American sample of 150 women with PTSD and depression secondary to IPV, researchers hypothesized that emotional flattening symptoms of depression could reduce threat detection and impede the termination of abusive relationships (Iverson et al. 2012). The authors tested this claim by investigating if reduced PTSD and depressive symptoms predicted less IPV re-victimization. They found that reducing PTSD and depressive symptoms through treatment led to significantly lower IPV re-victimization at 6-month follow-up (Iverson et al. 2012). Within the larger HAP trial, women who received HAP endorsed less IPV than counterparts enrolled in EUC at 12-month follow-up (Tol et al. 2019; Weobong et al. 2017). Although differences were not significant, the trend of higher IPV rates among women in EUC warrants future investigation of Iverson et al.’s (2012) hypothesis that treating depression can lower the risk of IPV re-victimization.

### Limitations

Failure to control for potential comorbidities (e.g., PTSD) is a limitation of this study. Our study also lacked a baseline or lifetime measure of IPV, attenuating our conclusions about the strength of post-treatment IPV as the sole predictor of treatment outcomes. Further, the length of marriage and timeframe of IPV exposure could have improved the understanding of how chronic IPV is experienced in this population. Our study did not explicitly examine the coping strategies or strengths of the participants. Our study is also limited by self-reported, rather than observed, activation. Finally, we did not conduct a priori power analyses to determine sample size requirements as we conducted secondary analyses. Therefore, we conducted a post hoc power analysis using G*Power software. Power was adequate for the HAP trial arm (power = 0.99), but analyses for the EUC trial arm were slightly underpowered (power = 0.70). Underpowered analyses may partly explain why the linear combination of predictors failed to meet significance in the EUC arm despite the individual predictor of IPV explaining significant variance in outcomes.

### Conclusions

Our findings provide preliminary support for reducing depressive symptoms among women experiencing IPV with culturally adapted BA. As trauma-focused care is contraindicated for individuals experiencing ongoing violence (Nickerson et al. 2011), improving one aspect of their health—depressive symptoms—through a brief treatment delivered by lay providers is promising in settings with prevalent IPV and depression.

## Electronic supplementary material

ESM 1 (DOCX 15 kb)
