# Supplementary material for: Psychological treatments for depression among women experiencing intimate partner violence: findings from a randomized controlled trial for behavioral activation in Goa, India
Source: Arch Womens Ment Health. 2019 Jul 30;22(6):779–89. doi: 10.1007/s00737-019-00992-2 (PMC6841649; doi:10.1007/s00737-019-00992-2)
Supplement: Supplementary file 1 — (DOCX 15 kb) [file 737_2019_992_MOESM1_ESM.docx]

Supplementary tables

Supplementary Table 1.

Correlation matrix of relevant variables for HAP, *r*^2^

|  | Baseline depressive symptoms | Age | 3-month depressive symptoms | 3-month PAAS | 12-month depressive symptoms | 12-month PAAS | 12-month IPV | 3-month IPV |
| --- | --- | --- | --- | --- | --- | --- | --- | --- |
| Baseline depressive symptoms | 1 | - | - | - | - | - | - | - |
| Age | 0.011 | 1 | - | - | - | - | - | - |
| 3-month depressive symptoms | 0.090 | -0.017 | 1 | - | - | - | - | - |
| 3-month PAAS | -0.054 | 0.090 | -.681^**^ | 1 | - | - | - | - |
| 12-month depressive symptoms | 0.088 | 0.156 | .415^**^ | -.450^**^ | 1 | - | - | - |
| 12-month PAAS | 0.047 | -0.064 | -.302^**^ | .530^**^ | -.587^**^ | 1 | - | - |
| 12-month IPV | 0.134 | -0.037 | .201^*^ | -0.110 | .214^*^ | -0.088 | 1 | - |
| 3-month IPV | -0.086 | -0.047 | 0.122 | -0.149 | 0.183 | -0.137 | .534^**^ | 1 |

*Note.* Pearson correlation, *r*, was used to assess correlations. PAAS = Premium Abbreviated Activation Scale, used to assess activation levels. Women’s education, occupation, and religion were not significantly correlated with the outcome variable and are therefore not unreported in this table.

^a^Baseline depressive symptoms was not normally distributed; the distribution was significantly positively skewed (higher depressive symptoms). Therefore, baseline depressive symptoms were logarithmically transformed to reduce positive skew; neither transformed nor original scores yielded significant correlations with variables of interest.
**Significant at the 0.01 level (2-tailed). *Significant at the 0.05 level (2-tailed).

Supplementary Table 2.

Correlation matrix of relevant variables for EUC, *r*^2^

| \|  \| Baseline depressive symptoms \| Age \| 3-month depressive symptoms \| 3-month PAAS \| 12-month depressive symptoms \| 12-month PAAS \| 12-month IPV \| 3-month IPV \| \| --- \| --- \| --- \| --- \| --- \| --- \| --- \| --- \| --- \| \| ^a^Baseline depressive symptoms \| 1 \| - \| - \| - \| - \| - \| - \| - \| \| Age \| 0.125 \| 1 \| - \| - \| - \| - \| - \| - \| \| 3-month depressive symptoms \| 0.088 \| -0.013 \| 1 \| - \| - \| - \| - \| - \| \| 3-month PAAS \| 0.001 \| .289^**^ \| -.436^**^ \| 1 \| - \| - \| - \| - \| \| 12-month depressive symptoms \| 0.092 \| 0.122 \| .464^**^ \| -0.146 \| 1 \| - \| - \| - \| \| 12-month PAAS \| -0.160 \| -0.051 \| -.431^**^ \| .377^**^ \| -.600^**^ \| 1 \| - \| - \| \| 12-month IPV \| -0.048 \| -0.114 \| .251^**^ \| -0.168 \| .268^**^ \| -.271^**^ \| 1 \| - \| \| 3-month IPV \| 0.071 \| -.214^*^ \| .277^**^ \| -0.130 \| .194^*^ \| -.268^**^ \| .499^**^ \| 1 \| | |
| --- | --- | --- | --- | --- | --- | --- | --- | --- | --- | --- | --- | --- | --- | --- | --- | --- | --- | --- | --- | --- | --- | --- | --- | --- | --- | --- | --- | --- | --- | --- | --- | --- | --- | --- | --- | --- | --- | --- | --- | --- | --- | --- | --- | --- | --- | --- | --- | --- | --- | --- | --- | --- | --- | --- | --- | --- | --- | --- | --- | --- | --- | --- | --- | --- | --- | --- | --- | --- | --- | --- | --- | --- | --- | --- | --- | --- | --- | --- | --- | --- | --- | --- |
| *Note.* Pearson correlation, *r*, was used to assess correlations. PAAS = Premium Abbreviated Activation Scale, used to assess activation levels. Women’s education, occupation, and religion were not significantly correlated with the outcome variable and are therefore not unreported in this table.  ^a^Baseline depressive symptoms was not normally distributed; the distribution was significantly positively skewed (higher depressive symptoms). Therefore, baseline depressive symptoms were logarithmically transformed to reduce positive skew; neither transformed nor original scores yielded significant correlations with variables of interest. **Significant at the 0.01 level (2-tailed). *Significant at the 0.05 level (2-tailed). |  |
